# Can Patients with Pancreatic Cancer and Liver Metastases Obtain Survival Benefit from Surgery? A Population-Based Study

**DOI:** 10.7150/jca.51218

**Published:** 2021-01-01

**Authors:** Bing-Bing Su, Dou-Sheng Bai, Jiang-Quan Yu, Chi Zhang, Sheng-Jie Jin, Bao-Huan Zhou, Guo-Qing Jiang

**Affiliations:** 1 Department of Hepatobiliary Surgery, Clinical Medical College, Yangzhou University, Yangzhou 225001, China.; 2 Department of Intensive Care Unit, Clinical Medical College, Yangzhou University, Yangzhou 225001, China.

**Keywords:** Pancreatic cancer, metastases, surgical procedures, survival, SEER

## Abstract

**Background:** Surgery for pancreatic cancer with liver metastases (PCL) is not recommended in the international guidelines, and investigation of its clinical significance in patients with PCL is very limited. This study explored whether surgery, especially synchronous resection of the primary tumor and liver metastases (SPL), could improve survival in PCL.

**Methods:** Data of 14,248 patients with PCL from Surveillance, Epidemiology, and End Results database was analyzed. Patients were divided into following groups: SPL, synchronous primary site, and other resection (SPO), single resection of the primary site (SPS), and no resection (NR).

**Results:** In this study, only 93 (0.7%) underwent SPL, 88 (0.6%) for SPO, and 232 (1.6%) for SPS. Multivariate Cox analysis showed surgical procedures of both the primary site and other sites were independent protective prognostic factors for pancreatic cancer cause-specific survival (PCSS) (all *P* < 0.001). Patients in the SPL group showed the most survival benefit, with a significant and gradually increased difference as compared with the SPO, SPS, and NR groups (median survival: 54, 34, 15, and 3 months, respectively, all *P* < 0.001). Compared with the NR group, mortalities were significant and gradually declining in the SPS, SPO, and SPL groups, with hazard ratio 0.329 (95% confidence interval [CI], 0.281 to 0.386), 0.220 (95% CI, 0.164 to 0.294), and 0.162 (95% CI, 0.118 to 0.222), respectively (all *P* < 0.001).

**Conclusions:** Surgical procedures for both primary site and other sites improved survival. SPL, particularly, showed a considerable survival benefit in well-selected patients with PCL.

## Introduction

Pancreatic cancer (PC) remains one of the most aggressive malignant tumors. Although the mortality from most cancers is declining, PC moved from the fourth leading cause of cancer-related death to the third in 2016 1. Despite of many efforts, this rate has not improved much over the last 30 years, with a persistently low 5-year survival rate of 8% 2,3. Compared with stages I-III, the overall survival (OS) of patients with stage IV metastatic disease (M1) was worse 4-6. The median survival of locally advanced PC is only 6-10 months, and just 3-6 months in M1 PC 7. Surgery is regarded as the only potentially curative method. However, once distant metastases are identified, surgery is not recommended in the guidelines 8,9.

For other malignant tumors, such as colorectal cancer, gastric cancer, and even sarcoma, there is increasing evidence that simultaneous metastasectomy can improve survival in appropriately selected patients who are in good general health and if the surgical procedures are performed carefully 10-12. The question arises whether all patients with M1 PC should face the presently dismal outcomes. Yet, it remains controversial whether there is a survival benefit from synchronous resection of both the primary tumor as well as metastases in patients with M1 PC.

A few studies including data from six European pancreas centers have all shown a significant survival benefit, with acceptable morbidity and mortality in patients with PC and liver metastases (PCL) who underwent synchronous resection of the primary tumor and liver metastases (SPL), in comparison with patients with PCL who did not undergo resection 13,14. Conversely, other studies have found no significant difference in survival between patients with PCL who underwent SPL and palliative bypass alone 15,16.

Up to the present, the sample sizes of patients undergoing SPL in previous studies have all been very small 13-16, with the largest sample including 69 patients in a collaboration study of six high-volume centers in Europe 14. To reach more robust conclusions, the present study aimed to use data from a larger patient sample to investigate the clinical significance of surgery, especially SPL, in patients with PCL. We extracted data from the Surveillance, Epidemiology, and End Results (SEER) cancer registry to systematically analyze the effect of surgery, especially SPL, on PC cause-specific survival (PCSS) in patients with PCL.

## Materials and Methods

### Patient selection in the SEER database

The SEER Cancer Statistics Review, which comprises the most recent statistics on cancer incidence, mortality, survival, prevalence, and lifetime risk, is published annually by the Data Analysis and Interpretation Branch of the National Cancer Institute in the United States (US). The current SEER database derives from 18 population-based cancer registries in the US 17. It contains no identifiers and is publicly available for studies of cancer-based epidemiology. We used SEER*Stat 8.3.5 software to identify patients with a histopathologic diagnosis of PC from January 1, 2010, through December 31, 2015, with follow-up through December 31, 2017.

SEER registry patients with PC who were eligible for our study cohort included those with the following histologic type, according to the International Classification of Diseases for Oncology, Third Edition: adenocarcinoma (8140, 8141, 8144, 8210, 8211, 8255, 8260, 8263, 8310, 8401, 8440, 8450, 8470, 8480, 8481, 8503, 8574, 8576), neuroendocrine carcinoma (8246) and others (8000, 8001, 8004, 8010, 8012, 8013, 8014, 8020, 8021, 8022, 8031, 8032, 8033, 8035, 8041, 8046, 8070, 8071, 8072, 8120, 8150, 8151, 8152, 8153, 8154, 8160, 8162, 8170, 8240, 8244, 8249, 8430, 8452, 8453, 8490, 8500, 8507, 8523, 8550, 8560, and 8980).

We extracted the following data: sex, race, age at diagnosis, year of diagnosis, primary site, pathological grade, histologic type, T stage, N stage, tumor size, insurance status, marital status, county percentage with a bachelor's degree, county percentage unemployed, county-level median household income, residential area, surgical procedure for the primary site, surgical procedure for other sites, radiotherapy, chemotherapy, SEER cause-specific death classification, SEER other cause of death classification, survival months, and vital status.

In this analysis, we included only adult patients with PC and liver metastases, with TNM stage IV, according to the criteria described in the American Joint Committee on Cancer Staging Manual (7th edition). We excluded patients as follows: those with bone metastasis, lung metastasis, brain metastasis, other primary cancer, unknown surgical history, unknown bachelor's degree status, and cause of death missing/unknown or attributable to causes other than PC.

### Statistical analysis

The primary endpoint of this study was PCSS. PCSS was defined as the time from the date of diagnosis to the date of death owing to PC. Baseline patient demographics and tumor characteristics were compared using the chi-square test. The PC survival rate was compared between subgroups using Kaplan-Meier analysis. All prognostic factors with *P* < 0.1 in Kaplan-Meier analysis were investigated using multivariate Cox analysis to identify predictors of PCSS. All statistical analyses were performed using IBM SPSS, version 22 (IBM Corp, Armonk, NY, USA). Statistical significance was set at two-sided *P* <0.05.

All patients were categorized as those receiving surgery for the primary site (PSP), those who were recommended but did not undergo surgery for the primary site (RN-PSP) group, and those who were not recommended and did not have surgery for the primary site (NRN-PSP). The PSP group was divided into the SPL group, synchronous primary tumor, and other resection (SPO) groups, and no synchronous resection for other sites group also called single resection of the primary site (SPS). A surgical procedure of other sites was defined as any of the following: (1) non-primary surgical procedure for liver; (2) non-primary surgical procedure for other regional sites; (3) non-primary surgical procedure for distant lymph node(s); (4) any combination of surgical procedures for other regional sites, distant lymph node(s), and/or liver; and (5) non-primary surgical procedure performed without detail information. Apart from non-primary surgical procedures for the liver, the remaining surgical procedures for other sites were defined as other resection.

## Results

### Baseline patient characteristics

We identified a total of 14,248 eligible patients with PCL between 2010 and 2015, with 7,711 male and 6,537 female patients. Of these, 93 (0.7%) underwent SPL, 88 (0.6%) received SPO, 232 (1.6%) received SPS, 414 (2.9%) PSP, 320 (2.3%) RN-PSP, 13,514 (94.8%) NRN-PSP and 13,503 (94.8%) patients received no resection (NR). Mean ages of patients were 58.5 ± 12.4 (range: 25-82) years in the SPL group, 55.7 ± 13.2 (range: 20-87) years in the SPO group, 60.6 ± 12.5 (range: 20-93) years in the SPS group, and 67.5 ± 12.1 (range: 20-103) years in the NR group.

In within-group comparisons, the SPL group had the highest proportion (53.8%) of body/tail site, greater frequency (36.0%) of well/moderately differentiated pathology grade, highest prevalence (41.9%) of neuroendocrine carcinoma, a greater proportion (72.0%) of T3 stage, and less (33.3%) chemotherapy, which were all statistically significant (P < 0.001). Baseline patient demographics and tumor characteristics according to different surgical procedures are described in **Table 1.**

### Effect on PCSS of surgical procedures for primary and other sites

Patients who underwent PSP had better survival (n = 13,834, 97.1%) than those who did not undergo surgery for the primary site (5-year PCSS: 33.4% vs. 0.19%, median survival: 24 vs. 3 months, *P* < 0.001). Five-year PCSS was 33.4% in the PSP group, 4.0% in the RN-PSP group, and 1.8% in the NRN-PSP group; survival was significantly different in Kaplan-Meier analysis (median survival: 24, 2, 3 months, respectively, *P* < 0.001). Surprisingly, the median survival of the RN-PSP group was significantly shorter than that of the NRN-PSP group (*P* < 0.001). Moreover, for surgical procedures of other sites, 5-year PCSS was 17.5% in the liver resection group, 16.9% in the other resection group, and 2.4% in the NR group; survival was also significantly different in Kaplan-Meier analysis (median survival: 8, 11, 3 months, respectively, *P* < 0.001).

As shown in** Table 2**, after univariate analysis and multivariate Cox analysis, surgical procedures of the primary site, surgical procedures of other sites, radiotherapy, and chemotherapy were all validated as independent protective prognostic factors for survival (all *P* < 0.001).

### Effect on PCSS of synchronous surgical procedure for primary and other sites

Among patients with PSP, only one had unknown surgery status for other sites; this patient was omitted from the following analyses. The 181 (43.8%) patients who received surgical procedures for other sites had better survival than the 232 (56.2%) patients who did not (5-year PCSS: 44.5% vs. 24.6%, median survival: 43 vs. 15 months, *P* < 0.001). As shown in **Table 3**, after univariate and multivariate Cox analyses, the synchronous surgical procedure of other sites was validated as an independent prognostic positive factor for survival (*P* < 0.001). Notably, radiotherapy and chemotherapy were not independent prognostic factors for survival in patients with PSP (**Table 3**).

### Effect of radiotherapy/chemotherapy on PCSS in patients without surgery

The 367 (2.7%) patients without surgery who received radiotherapy (WSR) had better survival than the 13,136 (97.3%) patients without surgery who received no/unknown radiotherapy (N-WSR) (2-year PCSS: 11.2% vs. 5.5%, median survival: 6 vs. 2 months, *P* < 0.001) (**Table 4**). The 6671 (49.4%) patients without surgery who received chemotherapy (WSC) had better survival than the 6832 (50.6%) patients without surgery who received no/unknown chemotherapy (N-WSC) (2-year PCSS: 8.4% vs. 3.0%, median survival: 6 vs. 1 months, *P* < 0.001) (**Table 4**).

As shown in **Table 4**, after univariate analysis and multivariate Cox analyses, radiotherapy and chemotherapy were validated as independent positive predictors of survival in patients without surgery (all *P* < 0.001).

### Subgroup analysis of the effect on PCSS of surgical procedures for the primary site, according to the primary site

As shown in **Table 5**, Kaplan-Meier analysis and multivariate Cox analyses showed that at each primary site, including the pancreatic head, body/tail, and other sites, patients receiving PSP had better survival than those receiving RN-PSP and NRN-PSP (all *P* < 0.001).

### Subgroup analysis of the effect on PCSS of surgical procedures for other sites, according to the primary site

As shown in **Table 5**, Kaplan-Meier and multivariate Cox analyses showed that at each primary site, including the pancreatic head, body/tail, and other sites, patients receiving NR had a worse survival than those in the liver resection and other resection groups (all *P* < 0.001).

### Subgroup analysis of the effect on PCSS of radiotherapy in patients without surgery, according to the primary site

As shown in **Table 5**, Kaplan-Meier and multivariate Cox analyses all showed that at each primary site, including the pancreatic head, body/tail, and other sites, patients receiving WSR had better survival than those with N-WSR (all *P* < 0.001).

### Subgroup analysis of the effect on PCSS of chemotherapy in patients without surgery, according to the primary site

As shown in **Table 5**, Kaplan-Meier and multivariate Cox analyses all showed that at each primary site, including the pancreatic head, body/tail, and other sites, patients receiving WSC had better survival than those with N-WSC (all *P* < 0.001).

### Subgroup analysis of the effect on PCSS of histology

**Figure 1:**
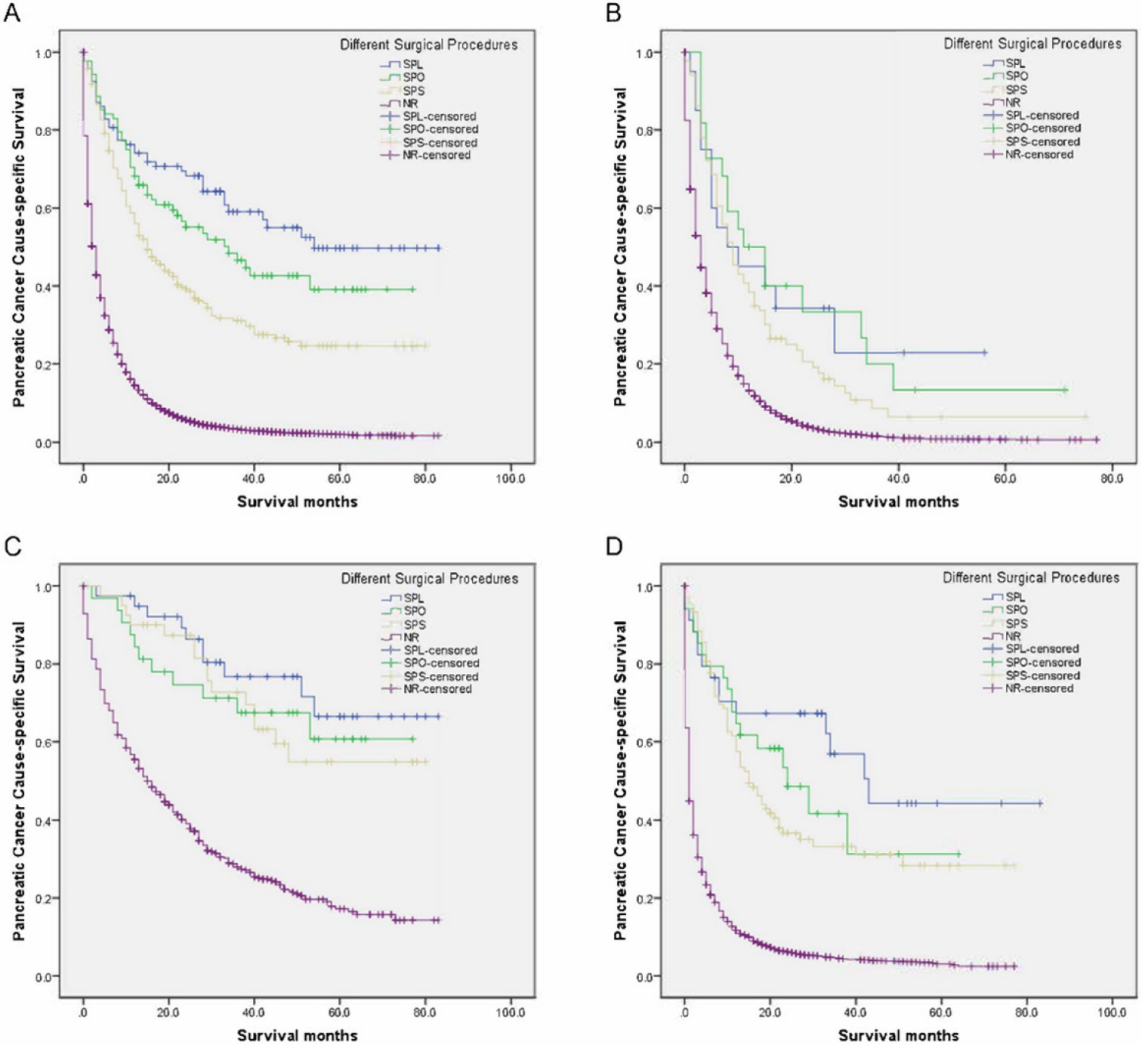
Survival curves in patients with pancreatic cancer and liver metastases treated with different surgical procedures. (A) Overall: χ^2^ = 113.429, *P* < 0.001; (B) Adenocarcinoma: Log rank χ^2^ = 84.148, *P* < 0.001; (C) Neuroendocrine carcinoma: Log rank χ^2^ = 74.889, *P* < 0.001; (D) Other: Log rank χ^2^ = 220.033, *P* < 0.001. SPL: synchronous resection of the primary tumor and liver metastases; SPO: synchronous primary tumor and other resection; SPS: single resection of the primary site; NR: no resection.

As seen in** Table 6**, the 5-year PCSS was significantly different and gradually declined in the following groups: 49.7% in the SPL group, 39.1% in the SPO group, 24.6% in the SPS group, and 1.9% in the NR group (*P* < 0.001) (**Figure 1A**). The SPS, SPO, and SPL groups showed significantly and gradually longer median survival of 15, 34, and 54 months, respectively, compared with 3 months for the NR group (all *P* < 0.001) (**Table 6**). Compared with the NR group, mortalities were significantly and gradually declining in the SPS, SPO, and SPL groups, with hazard ratio (HR) 0.329 (95% confidence interval [CI], 0.281-0.386), 0.220 (95% CI, 0.164-0.294), and 0.162 (95% CI, 0.118-0.222), respectively (all *P* < 0.001) (**Table 6**).

Compared with the NR group, there had increasingly improved survival benefits of 2-year PCSS for SPS, SPO, and SPL among adenocarcinoma, neuroendocrine carcinoma, or other groups (all *P* < 0.05) (**Table 6, Figure 1B-D**). Moreover, compared with the NR group, mortalities were significantly and gradually declining for SPS, SPO, and SPL among the adenocarcinoma, neuroendocrine carcinoma, or other groups (all *P* < 0.05) (**Table 6**).

**Figure 2:**
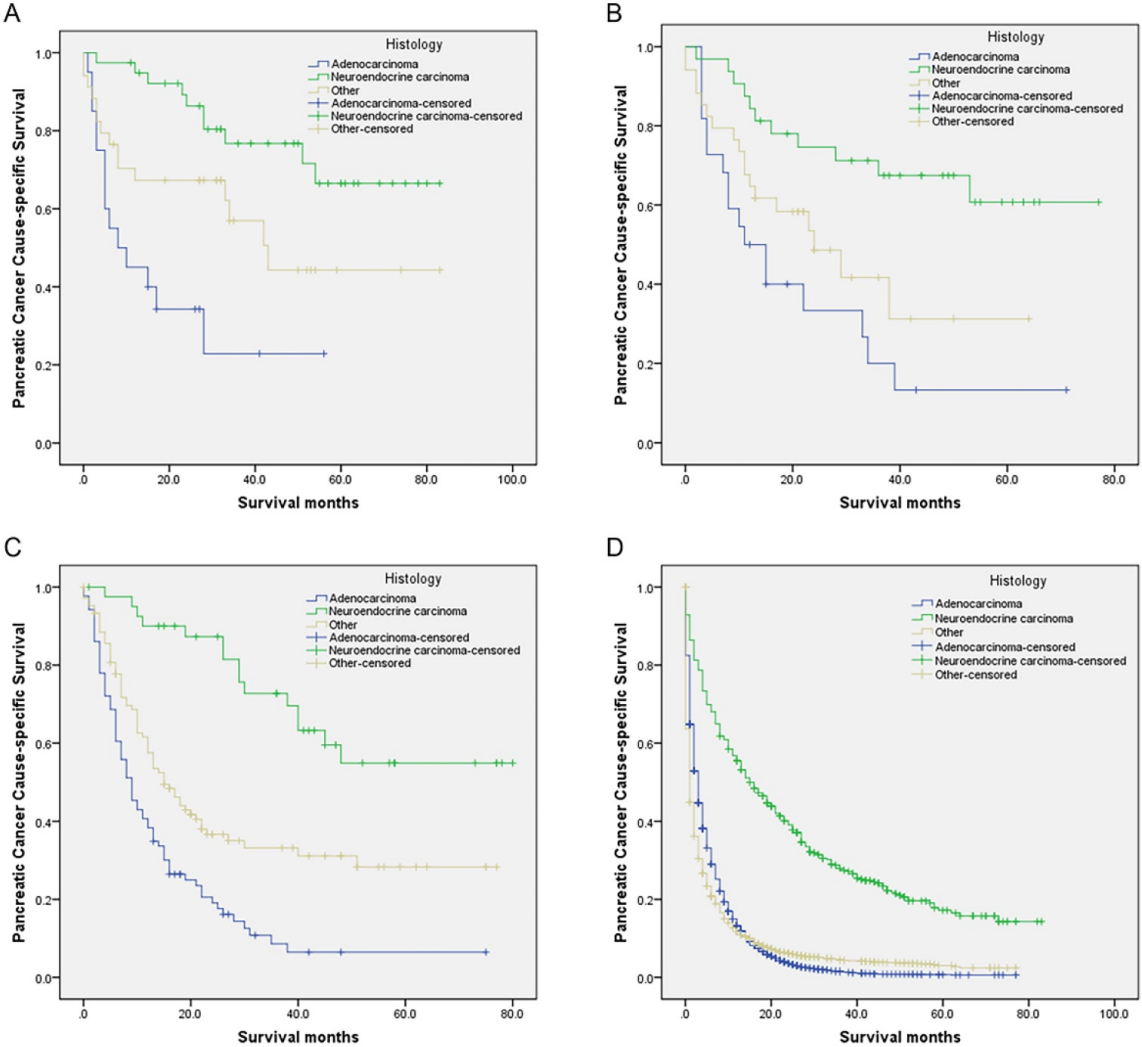
Survival curves in patients with pancreatic cancer and liver metastases treated with different histology. (A) SPL: χ2 = 19.873, *P* < 0.001; (B) SPO: Log rank χ^2^ = 14.658, *P* < 0.001; (C) SPS: Log rank χ^2^ = 47.873, *P* < 0.001; (D) NR: Log rank χ^2^ = 634.958, *P* < 0.001. Abbreviations: SPL: synchronous resection of the primary tumor and liver metastases; SPO: synchronous primary tumor and other resection; SPS: single resection of the primary site; NR: no resection.

Compared with the neuroendocrine carcinoma group, those who receiving SPS, SPO, SPL, or NR all had gradually worse PCSS for other histology and adenocarcinoma groups (all *P* < 0.05) (**Table 6, Figure 2A-D**). Moreover, compared with the neuroendocrine carcinoma group, mortalities were all significantly and gradually increased for other histology and adenocarcinoma groups receiving SPS, SPO, SPL, or NR (all *P* < 0.05) (**Table 6**).

### Subgroup analysis of the effect on PCSS of combined surgery and adjuvant therapy

Compared with patients receiving no/unknown adjuvant therapy, there were no significant differences in survival for chemoradiotherapy and chemotherapy with no/unknown radiotherapy among the SPL, SPO, or SPS groups in Kaplan-Meier and multivariate analyses with Cox regression (all *P* > 0.05) (**Table 6**).

Compared with patients receiving no/unknown adjuvant therapy, those with NR had increasingly improved survival benefits for radiotherapy with no/unknown chemotherapy, chemotherapy with no/unknown radiotherapy, and chemoradiotherapy (median survival: 1, 3, 6, and 8 months, respectively, all *P* < 0.001) (**Table 6**). Moreover, compared with patients receiving no/unknown adjuvant therapy, mortalities was significantly and gradually declining for the radiotherapy with no/unknown chemotherapy, chemotherapy with no/unknown radiotherapy, and chemoradiotherapy groups, with HR 0.569 (95% CI, 0.462-0.699, *P* < 0.001), 0.394 (95% CI, 0.379-0.408, *P* < 0.001), and 0.332 (95% CI, 0.292-0.377, *P* < 0.001), respectively (**Table 6**).

## Discussion

Current therapeutic approaches for patients with M1 PC are palliative and mainly based on tumor cell targeting. Some palliative chemotherapies' for patients with M1 PC have recently been established, such as the use of fluorouracil, leucovorin, irinotecan, and oxaliplatin (FOLFIRINOX) or gemcitabine with nab-paclitaxel, which have shown an increased median OS of 11 and 8.5 months, respectively, compared with 6.7-7 months for single gemcitabine 5; nevertheless, the survival outcome of patients with M1 PC remains poor.

Palliative resection for advanced pancreatic cancer is controversial. Tachezy et al. deemed that palliative resection for M1 PC was not advisable because of its lack of survival benefit (5.1 months [n = 22] vs. 5.8 months [n = 46]) and higher surgery-related morbidity (59% vs. 33%, P = 0.035) and mortality (27% vs. 7%, P = 0.049), compared with bypass surgery 18. Macroscopically complete resection has been demonstrated to be one of the most important and protective prognostic factors for survival; however, the performance of additional vessel resections and/or synchronous metastasis resections should be carefully weighed to avoid increasing morbidity and mortality caused by these surgical procedures 19-21.

International guidelines do not recommend surgery for PC when distant metastasis has occurred 1,9. Our outcomes showed that SPS was associated with significantly improved survival compared with no resection. The present rationale for proposing SPS in patients with PC and metastatic disease has been revisited in subgroup analyses. McKenzie et al. revealed significant survival benefits of 4.7 months in patients with M1 PC receiving SPS (median survival: 6.3 months, n = 92) compared with those who did not receive surgical resection (median survival: 1.6 months, n = 2606) 22.

Likewise, although synchronous resection for patients with PC and oligometastatic disease is controversial and not recommended in the international guidelines 1,9, with the increasing surgical safety of pancreatic and liver resection and unceasing pursuit for better survival in patients with M1 PC, SPL in carefully selected patients with PCL is being increasingly considered. Small studies, including case reports, have described the use of aggressive “curative” SPL in selected patients with PCL 10,23,24.

Two studies showed no survival benefit in PCL patients who underwent SPL, as compared with palliative bypass alone (median survival: 5.9 [n = 22] vs. 5.6 [n = 66] months; median survival: 6 [n = 11] vs. 4 [n = 22] months; all *P* > 0.05, respectively) 15,16.

Conversely, a previous study revealed significant survival benefits of 5.5 months in PCL patients who received SPL as compared with NR (median survival: 11.4 [n = 11] vs. 5.9 [n = 118] months; *P* = 0.0384) 13. A retrospective multicentral analysis in six European pancreas centers reported that the median OS of patients after SPL tended to be significantly longer than in those with NR (median survival: 14.5 [n = 69] vs. 7.5 [n = 69] months; *P* < 0.001) 14.

This study showed that surgical procedures of both the primary site and other sites were independent positive prognostic factors for survival. On the one hand, a good survival effect was seen in this study in that the SPS group had a 12-month increase in median survival compared with the NR group (*P* < 0.001). On the other hand, the median survival of the liver resection and other resection groups had 5- and 6-month increased survival in comparison with the NR group, respectively (all *P* < 0.001). Furthermore, regardless of whether the primary site was at the head, body/tail, or another location, resection of both the primary site and of other sites all significantly improved survival as compared with the NR group.

In this study, the SPL group showed the best survival benefit, with a significant and gradual increase in median survival of 20, 39, and 51 months, respectively, compared with the SPO, SPS, and NR groups (all *P* < 0.001). The mortality risk in the NR group was the highest, over six times that of the SPL group, nearly five times that of the SPO group, and over three times that of the SPS group. The median survival of the SPL group in this study was superior to that of the abovementioned studies 13-16. This difference may be owing to many factors including patients' performance status, surgical skills, perioperative management, management of operative indications, and in this study high rate of neuroendocrine carcinoma patients.

This study found that, among different histology groups, the neuroendocrine carcinoma group had the best survival for those who receiving SPS, SPO, SPL, or NR. On the contrary, the adenocarcinoma group had the worst survival. On the other hand, patients receiving SPL had a 29.7%, 42.4%, and 48.2% gradual increase in 2-year PCSS compared with whose receiving NR in adenocarcinoma, other histology, and neuroendocrine carcinoma groups respectively. In the study, we identified a total of 683 eligible PCL patients with neuroendocrine carcinoma. Fortunately, some of them, 112 (16.4%) received surgery, 39 (5.7%) underwent SPL, 32 (4.7%) received SPO, and 41 (6.0%) had SPS. PCL patients with neuroendocrine carcinoma after SPS, SPO, SPL were associated with gradual improved 5-year PCSS (54.9%, 60.7%, and 66.5%, respectively).

Another interesting finding of our study is that survival also improved in the other resection group. The median survival of the SPO group showed a 31-month increase compared with that of the NR group (*P* < 0.001). This finding is similar to a report by Shrikhande et al. that synchronous resection of interaortocaval lymph nodes (n = 9) and peritoneal metastases (n = 9) showed 7- and 21.1-month increase of median survival, respectively, compared with NR (n = 118) 13. Because of improved survival owing to adjuvant therapy, this is recommended for patients who have PC with or without surgical resection in the international guidelines 1,9; however, it is not mentioned as a treatment regimen for patients with M1 PC who receive synchronous multivisceral resection. Furthermore, clinical studies concerning the curative effect of adjuvant therapy in patients with M1 PC who receive synchronous multivisceral resection is very limited. Reportedly, postoperative chemotherapy and radiotherapy have no apparent influence on survival in patients with M1 PC who undergo synchronous multivisceral resection 19,22. The conclusions of this study were consistent with the abovementioned outcomes; even chemoradiotherapy did not significantly prolong postoperative survival. It is worth investigating why the addition of adjuvant therapy in patients with M1 PC who receive synchronous multivisceral resection is not associated with improved prognosis.

We found that the RN-PSP group had an even worse survival than the NRN-PSP group. This may be owing to patients' heavy psychological burden, rejecting surgery, or a lack of palliative therapy.

Our study had several limitations. First, surgery-related morbidity and mortality are not included in the SEER database. Second, recurrence data were unknown. Third, the data for radiation or chemotherapy were denoted “No/Unknown”; this is somewhat unclear and means that in the analysis, we did not have a patient group that did not receive either therapy. Fourth, it is clear that all patients had liver metastasis without metastasis to other common sites, such as bone, lung, and brain; however, whether patients had an uncommon metastatic disease is unknown. Fifth, the sequence concerning chemotherapy and surgery was unavailable. Sixth, details of chemotherapy including medications and dosage were not provided. Finally, detailed information on liver metastases was unavailable, including tumor size, number, and site.

To our best knowledge, the sample sizes of patients with PCL who underwent SPL, SPO, and SPS in this study may be the largest to date. We revealed that surgical procedures of both the primary site and other sites were independent protective predictors for survival in patients with PCL. Among the different treatment regimens, SPL in particular provided a considerable survival benefit. Besides, adjuvant therapies were not associated with improved postoperative survival in patients with PCL.

According to recent evidence, several guiding principles should be followed when performing SPL in patients with PCL. Surgical procedures should be carried out at a high-volume PC center by a multi-disciplinary team including surgeons experienced in procedures involving the pancreas, liver, and so on; also, patients should have good performance status, no invasion of the adjacent vessels, and resectability in limited liver metastases.

Further studies may be required, to develop qualification criteria for which PC center is qualified to perform SPL and operative indications for which patients with PCL are appropriate for SPL. In this population-based study, among 14,248 patients with PCL, only 93 (0.7%) received SPL, with a satisfactory 5-year PCSS (49.7%). In the future, it can be expected that increasingly more well-selected patients could benefit from SPL.

## Figures and Tables

**Table 1:** Baseline demographic and tumor characteristics of different surgical procedures for pancreatic cancer with liver metastases in the SEER database

Characteristic	SPL, N (%)(n = 93)	SPO, N (%)(n = 88)	SPS, N (%)(n = 232)	NR, N (%)(n = 13503)	*P*
**Sex**					0.525
Male	52 (55.9)	41 (46.6)	128 (55.2)	7312 (54.2)	
Female	41 (44.1)	47 (53.4)	104 (44.8)	6191 (45.8)	
**Race**					0.572
White	76 (81.7)	76 (86.4)	182 (78.4)	10608 (78.6)	
Black	10 (10.8)	6 (6.8)	30 (12.9)	1847 (13.7)	
Other*	7 (7.5)	6 (6.8)	20 (8.6)	1048 (7.8)	
**Age**					<0.001
<65	64 (68.8)	68 (77.3)	137 (59.1)	5552 (41.1)	
≥65	29 (31.2)	20 (22.7)	95 (40.9)	7951 (58.9)	
**Year of diagnosis**					0.539
2010-2011	26 (28.0)	27 (30.7)	69 (29.7)	4096 (30.3)	
2012-2013	29 (31.2)	36 (40.9)	84 (36.2)	4468 (33.1)	
2014-2015	38 (40.9)	25 (28.4)	79 (34.1)	4939 (36.6)	
**Primary Site**					<0.001
Head	32 (34.4)	25 (28.4)	123 (53.0)	4899 (36.3)	
Body/Tail	50 (53.8)	45 (51.1)	70 (30.2)	4856 (36.0)	
Other	11 (11.8)	18 (20.5)	39 (16.8)	3748 (27.8)	
**Grade**					<0.001
Well/Moderate	56 (60.2)	50 (56.8)	116 (50.0)	1040 (7.7)	
Poor/Anaplastic	21 (22.6)	28 (31.8)	71 (30.6)	1431 (10.6)	
Other	16 (17.2)	10 (11.4)	45 (19.4)	11032 (81.7)	
Histology					<0.001
Adenocarcinoma	20 (21.5)	22 (25.0)	86 (37.1)	9845 (72.9)	
Neuroendocrine carcinoma	39 (41.9)	32 (36.4)	41 (17.7)	546 (4.0)	
Other	34 (36.6)	34 (38.6)	105 (45.3)	3112 (23.0)	
**T stage**					<0.001
T0	0 (0.0)	0 (0.0)	0 (0.0)	119 (0.9)	
T1	4 (4.3)	3 (3.4)	10 (4.3)	337 (2.5)	
T2	17 (18.3)	15 (17.0)	41 (17.7)	3848 (28.5)	
T3	67 (72.0)	58 (65.9)	146 (62.9)	3280 (24.3)	
T4	4 (4.3)	10 (11.4)	16 (6.9)	2216 (16.4)	
TX	1 (1.1)	2 (2.3)	19 (8.2)	3703 (27.4)	
**N stage**					<0.001
N0	32 (34.4)	24 (27.3)	90 (38.8)	7173 (53.1)	
N1	60 (64.5)	60 (68.2)	132 (56.9)	3904 (28.9)	
NX	1 (1.1)	4 (4.5)	10 (4.3)	2426 (18.0)	
**Tumor Size**					<0.001
≤2 cm	7 (7.5)	5 (5.7)	19 (8.2)	714 (5.3)	
2-4 cm	37 (39.8)	34 (38.6)	99 (42.7)	4600 (34.1)	
>4 cm	45 (48.4)	45 (51.1)	98 (42.2)	5367 (39.7)	
Unknown	4 (4.3)	4 (4.5)	16 (6.9)	2822 (20.9)	
**Insurance status**					0.073
Insured	82 (88.2)	73 (83.0)	204 (87.9)	10999 (81.5)	
Medicaid	7 (7.5)	9 (10.2)	21 (9.1)	1790 (13.3)	
Uninsured/Unknown	4 (4.3)	6 (6.8)	7 (3.0)	714 (5.3)	
**Marital status**					<0.001
Married	59 (63.4)	52 (59.1)	156 (67.2)	7210 (53.4)	
Unmarried	30 (32.3)	32 (36.4)	66 (28.4)	5662 (41.9)	
Unknown	4 (4.3)	4 (4.5)	10 (4.3)	631 (4.7)	
**County % with bachelor degree**					0.511
Below median	37 (39.8)	40 (45.5)	90 (38.8)	5815 (43.1)	
Above median	56 (60.2)	48 (54.5)	142 (61.2)	7688 (56.9)	
**County % with unemployed**					0.779
Below median	40 (43.0)	43 (48.9)	107 (46.1)	6443 (47.7)	
Above median	53 (57.0)	45 (51.1)	125 (53.9)	7060 (52.3)	
**County-level median household income**					0.039
Below median	41 (44.1)	41 (46.6)	91 (39.2)	6532 (48.4)	
Above median	52 (55.9)	47 (53.4)	141 (60.8)	6971 (51.6)	
**Residence area**					0.301
Metropolitan	88 (94.6)	80 (90.9)	215 (92.7)	12054 (89.3)	
Urban/rural	5 (5.4)	8 (9.1)	17 (7.3)	1433 (10.6)	
Missing	0 (0.0)	0 (0.0)	0 (0.0)	16 (0.1)	
**Radiotherapy**					<0.001
Yes	7 (7.5)	8 (9.1)	11 (4.7)	367 (2.7)	
No/Unknown	86 (92.5)	80 (90.9)	221 (95.3)	13136 (97.3)	
**Chemotherapy**					<0.001
Yes	31 (33.3)	49 (55.7)	133 (57.3)	6671 (49.4)	
No/Unknown	62 (66.7)	39 (44.3)	99 (42.7)	6832 (50.6)	

*, Other includes American Indian/Alaska Native, Asian/Pacific Islander, and unknown. SPL: synchronous resection of the primary tumor and liver metastases; SPO: synchronous primary tumor and other resection; SPS: single resection of the primary site; NR: no resection.

**Table 2:** Univariate and multivariate Cox analysis to identify predictors of pancreatic cancer cause-specific survival

Variable	Total (n=14248)	2-year PCSS	5-year PCSS	Univariate analysis	Multivariate Cox analysis	
				*P*	HR (95%CI)	*P*
**Sex**				0.392		NI
Male	7711	0.071	0.032			
Female	6537	0.070	0.026			
**Race**				<0.001		0.018
White	11204	0.073	0.030		Reference	
Black	1946	0.055	0.021		1.061 (1.008-1.116)	0.023
Other*	1098	0.073	0.032		0.954 (0.894-1.019)	0.161
**Age**				<0.001		<0.001
<65	5991	0.112	0.047		Reference	
≥65	8257	0.041	0.017		1.306 (1.260-1.355)	
**Year of diagnosis**				<0.001		<0.001
2010-2011	4335	0.059	0.22		Reference	
2012-2013	4729	0.076	NA		0.955 (0.916-0.997)	0.034
2014-2015	5184	0.076	NA		0.915 (0.878-0.955)	<0.001
**Primary Site**				<0.001		0.016
Head	5236	0.065	0.024		Reference	
Body/Tail	5113	0.080	0.036		1.054 (1.012-1.098)	0.011
Other	3899	0.066	0.028		1.045 (0.998-1.094)	0.020
**Grade**				<0.001		<0.001
Well/Moderate	1301	0.252	0.126		Reference	
Poor/Anaplastic	1592	0.055	0.021		1.776 (1.639-1.925)	<0.001
Other	11355	0.052	0.019		1.525 (1.427-1.630)	<0.001
**Histology**				<0.001		<0.001
Adenocarcinoma	10248	0.039	0.008		Reference	
Neuroendocrine carcinoma	683	0.294	0.247		0.292 (0.265-0.322)	<0.001
Other	3317	0.084	0.047		0.893 (0.855-0.931)	<0.001
**T stage**				<0.001		0.004
T0	123	0.065	NA		Reference	
T1	360	0.092	0.038		0.829 (0.670-10.26)	0.085
T2	3993	0.069	0.029		0.819 (0.654-1.026)	0.082
T3	3656	0.098	0.041		0.769 (0.615-0.961)	0.021
T4	2314	0.060	0.023		0.781 (0.624-0.977)	0.031
TX	3802	0.052	0.021		0.838 (0.670-1.046)	0.118
**N stage**				<0.001		0.100
N0	7514	0.070	0.026		Reference	
N1	4257	0.084	0.041		1.044 (1.003-1.087)	0.034
NX	2477	0.050	0.018		1.007 (0.958-1.058)	0.794
**Tumor size**				<0.001		<0.001
≤2 cm	765	0.087	0.041		Reference	
2-4 cm	4890	0.072	0.024		1.093 (0.962-1.242)	0.170
>4 cm	5664	0.076	0.036		1.214 (1.069-1.379)	0.003
Unknown	2929	0.054	0.020		1.185 (1.040-1.350)	0.011
**Insurance status**				<0.001		<0.001
Insured	11627	0.075	0.032		Reference	
Medicaid	1871	0.049	0.021		1.098 (1.041-1.157)	<0.001
Uninsured/Unknown	750	0.051	0.010		1.181 (1.091-1.278)	<0.001
**Marital status**				<0.001		<0.001
Married	7658	0.088	0.035		Reference	
Unmarried	5920	0.048	0.020		1.122 (1.081-1.163)	<0.001
Unknown	670	0.080	0.044		0.982 (0.904-1.067)	0.671
**County % with bachelor degree**				<0.001		0.002
Below median	6146	0.066	0.024		Reference	
Above median	8102	0.075	0.034		0.939 (0.902-0.977)	
**County % with unemployed**				0.007		0.738
Below median	6788	0.076	0.034		Reference	
Above median	7460	0.066	0.025		0.994 (0.958-1.031)	
**County-level median household income**				<0.001		0.110
Below median	6905	0.064	0.024		Reference	
Above median	7343	0.077	0.034		0.967 (0.929-1.007)	
**Residence area**				0.183		NI
Metropolitan	12718	0.072	0.028			
Urban/rural	1514	0.063	0.035			
Missing	16	0.125	NA			
**Surgical procedure of primary site**				<0.001		<0.001
Not recommended	13514	0.058	0.018		Reference	
Performed	414	0.492	0.334		0.390 (0.339-0.448)	<0.001
Recommended but not Performed	320	0.049	0.040		0.910 (0.811-1.021)	0.107
**Surgical procedure of other sites**				<0.001		<0.001
No resection	13735	0.062	0.024		Reference	
Liver resection	288	0.298	0.175		0.714 (0.622-0.818)	<0.001
Other resection	218	0.286	0.169		0.772 (0.660-0.904)	0.001
Unknown	7	NA	NA		1.261 (0.599-2.652)	0.541
**Radiotherapy**				<0.001		<0.001
Yes	405	0.132	0.027		Reference	
No/Unknown	13843	0.069	0.029		1.303 (1.174-1.447)	
**Chemotherapy**				<0.001		<0.001
Yes	7079	0.096	0.030		Reference	
No/Unknown	7169	0.046	0.028		2.477 (2.384-2.573)	

*, Other includes American Indian/Alaska Native, Asian/Pacific Islander, and unknown. PCSS: pancreatic cancer cause-specific survival; HR: hazard ratio; CI: confidence interval; NA: not applicable; NI: not included in multivariate survival analysis.

**Table 3:** Univariate and multivariate Cox analyses to identify predictors of pancreatic cancer cause-specific survival in patients undergoing surgical procedures of the primary site

Variable	Total (n=413)	2-year PCSS	5-year PCSS	Univariate analysis	Multivariate Cox analysis	
				*P*	HR (95%CI)	*P*
**Sex**				0.858		NI
Male	221	0.050	0.344			
Female	192	0.485	0.324			
**Race**				0.782		NI
White	334	0.494	0.334			
Black	46	0.485	0.304			
Other*	33	0.498	0.409			
**Age**				<0.001		0.014
<65	269	0.579	0.405		Reference	
≥65	144	0.335	0.210		1.406 (1.071-1.846)	
**Year of diagnosis**				0.394		NI
2010-2011	122	0.466	0.287			
2012-2013	149	0.499	NA			
2014-2015	142	0.515	NA			
**Primary Site**				<0.001		0.019
Head	180	0.324	0.210		Reference	
Body/Tail	165	0.633	0.405		0.697 (0.503-0.967)	0.031
Other	68	0.586	0.487		0.566 (0.357-0.897)	0.016
**Grade**				<0.001		<0.001
Well/Moderate	222	0.673	0.476		Reference	
Poor/Anaplastic	120	0.272	0.174		2.425 (1.774-3.313)	<0.001
Other	71	0.320	0.188		1.772 (1.190-2.639)	0.005
**Histology**				<0.001		<0.001
Adenocarcinoma	128	0.237	0.097		Reference	
Neuroendocrine carcinoma	112	0.833	0.457		0.252 (0.161-0.394)	<0.001
Other	173	0.691	0.328		0.541 (0.392-0.747)	<0.001
**T stage**				0.002		0.471
T0	0	NA	NA			
T1	17	0.635	0.635		Reference	
T2	73	0.478	0.298		1.054 (0.330-3.362)	0.930
T3	271	0.512	0.352		1.026 (0.336-3.134)	0.965
T4	30	0.527	0.287		0.926 (0.273-3.145)	0.902
TX	22	0.156	0.104		2.195 (0.578-8.339)	0.248
**N stage**				0.067		0.162
N0	146	0.505	0.357		Reference	
N1	252	0.501	0.332		1.174 (0.864-1.597)	0.305
NX	15	0.240	0.160		1.870 (0.940-3.721)	0.075
**Tumor Size**				0.001		0.343
≤2 cm	31	0.562	0.515		Reference	
2-4 cm	170	0.430	0.280		1.866 (0.885-3.934)	0.101
>4 cm	188	0.573	0.384		1.863 (0.869-3.996)	0.110
Unknown	24	0.231	0.116		1.254 (0.460-3.421)	0.658
**Insurance status**				0.236		NI
Insured	359	0.505	0.347			
Medicaid	37	0.392	0.281			
Uninsured/Unknown	17	0.463	NA			
**Marital status**				0.051		0.041
Married	267	0.497	0.340		Reference	
Unmarried	128	0.448	0.281		1.173 (0.885-1.556)	0.267
Unknown	18	0.769	0.684		0.351 (0.134-0.922)	0.034
**County % with bachelor degree**				0.042		0.080
Below median	167	0.448	0.285		Reference	
Above median	246	0.524	0.371		0.767 (0.570-1.033)	
**County % with unemployed**				0.040		0.439
Below median	190	0.522	0.409		Reference	
Above median	223	0.468	0.268		1.129 (0.830-1.537)	
**County-level median household income**				0.002		0.034
Below median	173	0.428	0.268		Reference	
Above median	240	0.540	0.386		0.709 (0.516-0.975)	
**Residence area**				0.104		NI
Metropolitan	383	0.507	0.339			
Urban/rural	30	0.323	0.277			
**Synchronous surgical procedure**				<0.001		0.011
SPS	232	0.392	0.246		Reference	
SPL	93	0.683	0.497		0.544 (0.373-0.793)	0.009
SPO	88	0.551	0.391		0.656 (0.461-0.934)	0.033
**Radiotherapy**				0.513		NI
Yes	26	0.498	0.249			
No/Unknown	387	0.491	0.337			
**Chemotherapy**				0.001		0.056
Yes	213	0.414	0.228		Reference	
No/Unknown	200	0.580	0.451		1.365 (0.992-1.878)	

*, Other includes American Indian/Alaska Native, Asian/Pacific Islander, and unknown. PCSS: pancreatic cancer cause-specific survival; HR: hazard ratio; CI: confidence interval; SPL: synchronous resection of the primary tumor and liver metastases; SPO: synchronous primary tumor and other resection; SPS: single resection of the primary site; NA: not applicable; NI: not included in multivariate survival analysis.

**Table 4:** Univariate and multivariate Cox analyses to identify predictors of pancreatic cancer cause-specific survival in patients receiving no resection

Variable	Total (n=13503)	2-year PCSS	5-year PCSS	Univariate analysis	Multivariate Cox analysis	
				*P*	HR (95%CI)	*P*
**Sex**				0.298		NI
Male	7312	0.057	0.022			
Female	6191	0.056	0.016			
**Race**				0.004		0.019
White	10608	0.059	0.021		Reference	
Black	1847	0.045	0.013		1.059 (1.006-1.116)	0.030
Other*	1048	0.059	0.019		0.950 (0.889-1.016)	0.133
**Age**				<0.001		<0.001
<65	5552	0.088	0.029		Reference	
≥65	7951	0.035	0.013		1.295 (1.247-1.344)	
**Year of diagnosis**				<0.001		0.001
2010-2011	4096	0.046	0.014		Reference	
2012-2013	4468	0.061	NA		0.962 (0.921-1.004)	0.077
2014-2015	4939	0.062	NA		0.922 (0.883-0.963)	<0.001
**Primary Site**				<0.001		0.004
Head	4899	0.054	0.016		Reference	
Body/Tail	4856	0.061	0.023		1.062 (1.019-1.107)	0.005
Other	3748	0.050	0.019		1.069 (1.020-1.121)	0.005
**Grade**				<0.001		<0.001
Well/Moderate	1040	0.163	0.055		Reference	
Poor/Anaplastic	1431	0.034	0.008		1.708 (1.569-1.859)	<0.001
Other	11032	0.050	0.018		1.476 (1.379-1.581)	<0.001
**Histology**				<0.001		<0.001
Adenocarcinoma	9845	0.036	0.007		Reference	
Neuroendocrine carcinoma	546	0.391	0.062		0.298 (0.269-0.330)	<0.001
Other	3112	0.172	0.030		0.911(0.872-0.951)	<0.001
**T stage**				<0.001		0.006
T0	119	0.060	NA		Reference	
T1	337	0.064	0.012		0.855 (0.689-1.064)	0.156
T2	3848	0.059	0.025		0.809 (0.643-1.018)	0.070
T3	3280	0.063	0.016		0.761 (0.606-0.956)	0.019
T4	2216	0.052	0.018		0.769 (0.612-0.968)	0.025
TX	3703	0.050	0.021		0.829 (0.660-1.040)	0.106
**N stage**				<0.001		0.183
N0	7173	0.060	0.019		Reference	
N1	3904	0.056	0.022		1.037 (0.996-1.081)	0.079
NX	2426	0.048	0.017		0.998 (0.949-1.050)	0.950
**Tumor Size**				<0.001		<0.001
≤2 cm	714	0.065	0.021		Reference	
2-4 cm	4600	0.058	0.014		1.102 (0.966-1.258)	0.148
>4 cm	5367	0.058	0.024		1.228 (1.076-1.400)	0.002
Unknown	2822	0.052	0.020		1.183 (1.033-1.354)	0.015
**Insurance status**				<0.001		<0.001
Insured	10999	0.060	0.021		Reference	
Medicaid	1790	0.041	0.015		1.094 (1.036-1.154)	0.001
Uninsured/Unknown	714	0.043	0.007		1.164 (1.074-1.262)	<0.001
**Marital status**				<0.001		<0.001
Married	7210	0.071	0.024		Reference	
Unmarried	5662	0.038	0.014		1.116 (1.075-1.158)	<0.001
Unknown	631	0.061	0.025		1.000 (0.919-1.089)	0.997
**County % with bachelor degree**				<0.001		0.003
Below median	5815	0.053	0.016		Reference	
Above median	7688	0.060	0.023		0.941 (0.903-0.980)	
**County % with unemployed**				0.008		0.688
Below median	6443	0.062	0.023		Reference	
Above median	7060	0.052	0.017		0.992 (0.956-1.030)	
**County-level median household income**				<0.001		0.182
Below median	6532	0.053	0.018		Reference	
Above median	6971	0.060	0.022		0.972 (0.932-1.013)	
**Residence area**				0.696		NI
Metropolitan	12054	0.056	0.018			
Urban/rural	1433	0.058	0.030			
Missing	16	0.125	NA			
**Radiotherapy**				<0.001		<0.001
Yes	367	0.112	0.009		Reference	
No/Unknown	13136	0.055	0.020		1.330 (1.194-1.483)	
**Chemotherapy**				<0.001		<0.001
Yes	6671	0.084	0.024		Reference	
No/Unknown	6832	0.030	0.014		2.509 (2.413-2.608)	

*, Other includes American Indian/Alaska Native, Asian/Pacific Islander, and unknown. PCSS: pancreatic cancer cause-specific survival; HR: hazard ratio; CI: confidence interval; NA: not applicable; NI: not included in multivariate survival analysis.

**Table 5:** Univariate and multivariate Cox analyses of pancreatic cancer cause-specific survival according to primary site

Variable	Total	Median survival (months)	2-year PCSS	5-year PCSS	Univariate analysis	Multivariate Cox analysis	
					*P*	HR (95%CI)	*P*
***Surgical procedure of primary site***							
**Primary Site:**							
**Head**	5236	3			<0.001		<0.001
Performed	181	13	0.322	0.209		Reference	
Recommended but not performed	95	2	0.037	NA	<0.001	3.615 (2.759-4.737)	<0.001
Not recommended	4960	3	0.056	0.016	<0.001	2.681 (2.249-3.197)	<0.001
**Body/Tail**	5113	3			<0.001		<0.001
Performed	165	38	0.633	0.405		Reference	
Recommended but not performed	100	2	0.061	0.037	<0.001	5.749 (4.275-7.730)	<0.001
Not recommended	4848	3	0.061	0.022	<0.001	4.926 (3.952-6.141)	<0.001
**Other**	3899	2			<0.001		<0.001
Performed	68	53	0.586	0.487		Reference	
Recommended but not performed	125	1	0.050	NA	<0.001	5.872 (3.947-8.735)	<0.001
Not recommended	3706	2	0.057	0.019	<0.001	5.197 (3.644-7.414)	<0.001
***Surgical Procedure of Other Sites***							
**Primary Site:**							
**Head^a^**	5235	3			<0.001		<0.001
Not performed	5022	3	0.058	0.019		Reference	
Liver resection	125	8	0.239	0.140	<0.001	0.519 (0.426-0.633)	<0.001
Other resection	88	8	0.196	0.103	<0.001	0.549 (0.436-0.691)	<0.001
**Body/Tail^b^**	5111	3			<0.001		<0.001
Not performed	4926	3	0.069	0.028		Reference	
Liver resection	106	11	0.372	0.229	<0.001	0.395 (0.314-0.497)	<0.001
Other resection	79	13	0.382	0.245	<0.001	0.376 (0.287-0.492)	<0.001
**Other^c^**	3895	2			<0.001		<0.001
Not performed	3787	2	0.060	0.024		Reference	
Liver resection	57	7	0.283	0.136	<0.001	0.513 (0.383-0.687)	<0.001
Other resection	51	11	0.298	0.201	<0.001	0.413 (0.299-0.568)	<0.001
***Radiotherapy^d^***							
**Primary Site:**							
**Head**	4899	3			<0.001		<0.001
Yes	173	6	0.103	NA		Reference	
No/Unknown	4726	3	0.052	0.018		1.431 (1.225-1.673)	
**Body/Tail**	4856	3			<0.001		<0.001
Yes	111	5	0.122	0.030		Reference	
No/Unknown	4745	2	0.060	0.023		1.439 (1.184-1.750)	
**Other**	3748	2			<0.001		<0.001
Yes	83	8	0.121	NA		Reference	
No/Unknown	3665	2	0.053	0.019		1.736 (1.377-2.190)	
***Chemotherapy^d^***							
**Primary Site:**							
**Head**	4899	3			<0.001		<0.001
Yes	2453	7	0.077	0.018		Reference	
No/Unknown	2446	1	0.031	0.014		2.572 (2.422-2.731)	
**Body/Tail**	4856	3			<0.001		<0.001
Yes	2579	6	0.088	0.028		Reference	
No/Unknown	2277	1	0.031	0.017		2.540 (2.390-2.699)	
**Other**	3748	2			<0.001		<0.001
Yes	1639	6	0.089	0.029		Reference	
No/Unknown	2109	1	0.027	0.012		2.456 (2.291-2.632)	

^a^, Excluding one patient in whom surgical procedures of other sites was unknown. ^b^, Excluding two patients in whom surgical procedures of other sites was unknown. ^c^, Excluding four patients in whom surgical procedures of other sites was unknown. ^d^, Patients who did not undergo resection. PCSS: pancreatic cancer cause-specific survival; HR: hazard ratio; CI: confidence interval; NA: not applicable.

**Table 6:** Univariate and multivariate Cox analyses to evaluate pancreatic cancer cause-specific survival with histology and combined therapies

Variable	Total	Median survival (months)	2-year PCSS	5-year PCSS	Univariate analysis	Multivariate Cox analysis	
					*P*	HR (95%CI)	*P*
Total	13916	3			<0.001		<0.001
No resection	13503	3	0.057	0.019		Reference	
SPS	232	15	0.392	0.246	<0.001	0.329 (0.281-0.386)	<0.001
SPL	93	54	0.683	0.497	<0.001	0.162 (0.118-0.222)	<0.001
SPO	88	34	0.551	0.391	<0.001	0.220 (0.164-0.294)	<0.001
**Histology**							
Adenocarcinoma	9973	3			<0.001		<0.001
No resection	9845	3	0.036	0.006		Reference	
SPS	86	9	0.191	0.065	<0.001	0.495 (0.394-0.621)	<0.001
SPL	20	8	0.343	NA	<0.001	0.360 (0.215-0.614)	<0.001
SPO	22	11	0.333	0.133	<0.001	0.361 (0.224-0.581)	<0.001
**Neuroendocrine carcinoma**	658	21			<0.001		<0.001
No resection	546	15	0.391	0.172		Reference	
SPS	41	NA	0.863	0.549	<0.001	0.290 (0.173-0.486)	<0.001
SPL	39	NA	0.873	0.665	<0.001	0.193 (0.103-0.363)	<0.001
SPO	32	NA	0.746	0.607	<0.001	0.278 (0.152-0.506)	<0.001
**Other**	3285	1			<0.001		<0.001
No resection	3112	1	0.062	0.030		Reference	
SPS	105	15	0.367	0.283	<0.001	0.312 (0.244-0.398)	<0.001
SPL	34	43	0.673	0.443	<0.001	0.191 (0.115-0.318)	<0.001
SPO	34	24	0.486	0.312	<0.001	0.251 (0.158-0.400)	<0.001
SPS	232	15			0.472^a^		0.705
No/Unknown	97	15	0.420	0.352		Reference	
**Chemoradiotherapy**	9	18	0.444	NA	0.839	1.084 (0.493-2.382)	0.841
Chemotherapy*	124	15	0.362	0.178	0.423	1.152 (0.826-1.607)	0.403
Radiotherapy^#^	2	-	-	-			
SPL	93	54			0.182^b^		0.198
No/Unknown	60	NA	0.749	0.589		Reference	
**Chemoradiotherapy**	5	23	0.400	NA	0.615	1.480 (0.342-6.401)	0.600
Chemotherapy*	26	42	0.321	NA	0.071	1.838 (0.945-3.576)	0.073
Radiotherapy^#^	2	-	-	-			
SPO	88	33			0.340^c^		0.353
No/Unknown	38	53	0.673	0.438		Reference	
**Chemoradiotherapy**	7	34	0.536	0.357	0.662	1.266 (0.426-3.768)	0.671
Chemotherapy*	42	16	0.436	0.355	0.145	1.577 (0.849-2.929)	0.149
Radiotherapy^#^	1	-	-	-			
No resection	13503	3			<0.001		<0.001
No/Unknown	6736	1	0.029	0.015		Reference	
**Chemoradiotherapy**	271	8	0.119	0.011	<0.001	0.332 (0.292-0.377)	<0.001
Chemotherapy*	6400	6	0.083	0.025	<0.001	0.394 (0.379-0.408)	<0.001
Radiotherapy^#^	96	3	0.091	NA	<0.001	0.569 (0.462-0.699)	<0.001

*, No/unknown radiotherapy. ^#^, No/unknown chemotherapy. ^a^, Analysis did not include the radiotherapy group because there were only two patients who received radiotherapy. ^b^, Analysis did not include the radiotherapy group because there were only two patients who received radiotherapy. ^c^, Analysis did not include the radiotherapy group because there was only one patient who received radiotherapy. PCSS: pancreatic cancer cause-specific survival; HR: hazard ratio; CI: confidence interval; SPL: synchronous resection of the primary tumor and liver metastases; SPO: synchronous primary tumor and other resection; SPS: single resection of the primary site; NA: not applicable.

## Abbreviations

PC: pancreatic cancer
PCL: pancreatic cancer with liver metastases
SPL: synchronous resection of the primary tumor and liver metastases
SPO: synchronous primary tumor and other resection
SPS: single resection of the primary site
NR: no resection
PCSS: pancreatic cancer cause-specific survival
PSP: receiving surgery for the primary site
RN-PSP: recommended but did not undergo surgery for the primary site
NRN-PSP: not recommended and did not have surgery for the primary site
WSR: without surgery who received radiotherapy
N-WSR: without surgery who received no/unknown radiotherapy
WSC: without surgery who received chemotherapy
N-WSC: received no/unknown chemotherapy
SEER: Surveillance, Epidemiology, and End Results
HR: hazard ratio
CI: confidence interval
OS: overall survival
NA: not applicable
NI: not included in multivariate survival analysis
